# Zika virus can be venereally transmitted between *Aedes aegypti* mosquitoes

**DOI:** 10.1186/s13071-017-2543-4

**Published:** 2017-12-15

**Authors:** Stéphanie Silva Campos, Rosilainy Surubi Fernandes, Alexandre Araujo Cunha dos Santos, Rafaella Moraes de Miranda, Erich Loza Telleria, Anielly Ferreira-de-Brito, Marcia Gonçalves de Castro, Anna-Bella Failloux, Myrna C. Bonaldo, Ricardo Lourenço-de-Oliveira

**Affiliations:** 1 Laboratório de Mosquitos Transmissores de Hematozoários, Instituto Oswaldo Cruz, Fiocruz, Rio de Janeiro, Brazil; 2 Laboratório de Biologia Molecular de Flavivírus, Instituto Oswaldo Cruz, Fiocruz, Rio de Janeiro, Brazil; 3 Laboratório de Biologia Molecular de Parasitos e Vetores, Instituto Oswaldo Cruz, Fiocruz, Rio de Janeiro, Brazil; 4 Arboviruses and Insect Vectors, Institut Pasteur, Paris, France

**Keywords:** Zika virus, *Aedes aegypti*, Venereal transmission

## Abstract

**Background:**

Alternative transmission routes have been described for Zika virus (ZIKV). Here, we assessed for the first time the venereal transmission of ZIKV between *Aedes aegypti* under laboratory conditions*.*

**Results:**

Orally-infected mosquito females were able to transmit the virus to males venereally, and males inoculated intrathoracically were capable of infecting females during mating. The genome of venereally-transmitted virus recovered from males was identical to that of ZIKV ingested by mated females.

**Conclusion:**

We conclude that venereal transmission between *Aedes* mosquitoes might contribute to Zika virus maintenance in nature.

## Background

Zika virus (ZIKV, *Flavivirus*, *Flaviviridae*) is an arbovirus that has undergone a rapid spread in the Pacific region and across tropical America since 2015, causing a severe pandemic in 2015–2016 [1–3]. Moreover, microcephaly and other congenital neurological malformations and disorders were associated with ZIKV infections worldwide, which have made this virus currently one of the most significant public health issues. ZIKV transmission to people is primarily through the bite of an infected *Aedes* mosquitoes, mainly *Aedes aegypti* [1, 4]*.* However, the description of other modes of transmission, like inter-human contamination, may also explain ZIKV emergence, its efficient spread and maintenance in nature [5–8]. Moreover, inter-mosquito transmission through the vertical route is also likely to play a role. Therefore, we experimentally demonstrated that ZIKV could be venereally transmitted between *Ae. aegypti*, a phenomenon that could help in perpetuating ZIKV in nature.

## Methods

We experimentally assessed venereal transmission in two populations of Brazilian *Ae. aegypti* mosquitoes: Urca (URC; F2 generation), Rio de Janeiro, coastal Southeast region, and Goiânia (GOI; F1 generation), Goiás, inland Central-West region. Their F0 generation tested negative for ZIKV by qRT-PCR as described previously [9]. Mosquitoes were reared at 26 ± 1 °C, 12 h:12 h light:dark cycle and 70 ± 10% humidity (standardized environmental condition, SEC) as previously described [9, 10]. Mosquitoes were sexed at the pupal stage, and the emerged adults of each gender were maintained in distinct cages supplied with a 10% sucrose solution at SEC. Virgin *Ae. aegypti* females aged 5–7 days post-emergence were orally challenged with two ZIKV strains belonging to the Asian lineage, namely ZIKV-Rio-U1 (GenBank: KU926309) and ZIKV-PE243 (GenBank: KX197192), respectively, isolated from humans in Southeast [11] and Northeast [12] Brazil. These ZIKV strains display 99% nucleotide and amino acid identity and may similarly infect Brazilian *Ae. aegypti* [9–12]. Accordingly, females were provided with a mixture containing two parts of washed rabbit erythrocytes and one part of viral suspension at a final titter of 10^6^ PFU/ml as described previously [9]. We incubated sets of fully engorged females at SEC in cylindrical carton cages (20 × 16 cm) daily supplied with a 10% sucrose solution. Groups of 10–30 females corresponding to each combination of the mosquito population and virus strain were randomly killed, and body homogenates in culture medium were examined by RT-qPCR for determining infection rates (IR) on the 10th and 14th day post-infection (dpi) as described elsewhere [9]. At this same dpi, virgin males of the same population, generation and age were introduced into the respective female-infected cages at two sex ratios (Table 1). Then, distinct groups of mosquitoes were allowed to freely mate for 30 h devoid of any food source, to avoid oral contamination, or for 5 days provided with a piece of cotton mesh imbibed with 10% sucrose solution on the top of the cage. In the last case, samples of the sucrose solution probed by mosquitoes were examined daily for the presence of ZIKV by RT-qPCR, and have always tested ZIKV-negative. On completion of the above-mentioned contact periods, samples of 30 females were dissected to determine insemination rates, that is, the proportion of females with spermatheca containing spermatozoids [13]. RNA was extracted from male entire body homogenates for determining IR by RT-qPCR and whole viral genome sequencing on the 14th day after mating as described [9, 11]. Due to the scanty number of health or surviving males that mated with infected females, we decide to intrathoracically inject virgin males with ZIKV to access venereal transmission from male to female. Thus, the supernatant of the body homogenate of one venereally contaminated URC male was inoculated in other URC virgin males. After 10 and 14 days of incubation at SEC, URC virgin females were left to mate for 30 h (devoid of any food source) at a sex ratio of 1 male:2 females, and incubated for 14 days at SEC. Male and female entire body homogenates were tested by RT-qPCR [9]. Aiming to investigate possible genetic changes in the viral genome occurring during the venereal transmission, we also extracted RNA and sequenced the whole genome from the virus of the infectious meal taken by females as well as from a pool of five infected females that mated with the venereally contaminated males. Nucleotide sequences were aligned, edited and compared as described previously [11].

**Table 1:** Infection rates in *Aedes aegypti* females orally challenged with ZIKV and males contaminated by the venereal route

Mosquito population	ZIKV strain	Sex ratio female: male (insemination rate)^a^	Female orally challenged		Male venereal transmission	
			*n*	IR (%)	*n*	IR (%)
Urca	RioU1	256:256 (90%)^b^	30	63	110	0.9
	RioU1	37:19 (97%)^c^	10	70	20	0
	PE243	76:76 (50%)^b^	30	46	38	0
Goiânia	RioU1	90:45 (100%)^c^	10	50	6	33.0

*Abbreviations: n* number of tested mosquitoes, *IR* infection rate referring to the proportion of mosquitoes with infected body among tested ones

^a^Insemination rate is the proportion of females with spermathecal containing spermatozoids

^b^Contact for 30 h

^c^Contact for 5 days

## Results

Insemination rates were 50–90% and 100% in females kept in contact with males for 30 h and 5 days, respectively. IRs in orally challenged females ranged from 46% to 70% in URC mosquitoes challenged with ZIKV-PE243 and ZIKV-Rio-U1, respectively (Table 1). Venereal transmission to males was achieved in mosquito groups whose females were infected with ZIKV-Rio-U1, where IRs in males was 0.9% in URC and 33% in GOI (median of viral loads in bodies of 1.5 × 10^6^ RNA copies/ml; CT ≤ 17.2). No venereal transmission was detected in the mosquito groups infected with ZIKV-PE243, whose females coincidently had the lowest insemination rates. IRs of intrathoracically inoculated males were 87.5 and 97.9% at 10 and 14 days, respectively (Table 2). Viral load in bodies of males at 10 and 14 days after injection reached 3.6 × 10^6^ and 9.8 × 10^6^ RNA copies/ml (median: 3.3 × 10^6^ and 3.6 × 10^6^; CT ≤ 15.6), respectively. Males with 10 days after inoculation could not venereally transmit the virus. However, the venereal transmission was detected in 50% of females mated by males with 14 days after intrathoracic injection (viral load in bodies of positive females ranging from 3.4 × 10^2^ to 9.8 × 10^2^ RNA copies/ml; CT ≤ 28.8). The compared ZIKV genomes were identical, with the virus recovered from a venereally contaminated male (URC-ZIKVRioU1) presenting a single nucleotide substitution at position 1115 (protein E) in which a thymine was replaced by a cytosine, without generating amino acid change.

**Table 2:** Infection rates in *Aedes aegypti* males inoculated intrathoracically with ZIKV-RioU1 and female contaminated by the venereal route. Mosquitoes were let to copulate for 30 h devoid of any food source at a sex ratio of: 1 male: 2 females (81:162)

Days after intrathoracic inoculation of males	Male inoculated intrathoracically		Female venereal transmission	
	n	IR-mi (%)	n	IR-mi (%)
10	10	87.5	19	0
14	41	97.9	10	50

*Abbreviations: n* number of tested mosquitoes, *IR* Infection rate referring to the proportion of mosquitoes with infected body among tested ones by RT-qPCR

## Discussion

Male *Aedes* mosquitoes have been found naturally infected with ZIKV in Africa [14] and South America [4], providing evidence that inter-mosquito contamination occurs in nature and this phenomenon may play some role in virus maintenance and viral evolution [15]. Being non-hematophagous, male mosquitoes may acquire an arbovirus infection through the vertical route. Nevertheless, the venereal transmission has also been experimentally demonstrated for several pairings insect-arbovirus [15–17], including other flaviviruses such as Japanese encephalitis, dengue and St Louis encephalitis viruses [18–20]. We demonstrated that ZIKV could be venereally transmitted between *Ae. aegypti* of two geographically-distant populations. Males obtained the infection from females and vice versa. Moreover, the genome of the virus transmitted by the venereal route was identical to those orally absolved as well as infecting females, suggesting that no expressive genetic changes occur in the viral genome during the venereal transmission. The efficiency of the venereal transmission route may be greater than expected. The variation in insemination and infection rates in orally-challenged females suggest that not all of them had sexual contact with males, especially in the groups brought into contact for only 30 h. The higher rate of venereal transmission from infected females to males (33%) was detected in the group of mosquitoes in which contact time was 5 days and insemination rate was of 100%, that is, all females had sexual contact with males. Considering that one male could have inseminated more than one female in such a group [13], it is possible that not all males had sexual contact with infected females, reducing the chance of venereal transmission. These data altogether may explain the large variation in male IRs.

Several factors have been proposed to explain the silent endemic/enzootic maintenance of ZIKV in Africa and Asia as well as the recent fast spread of this virus in the West Hemisphere, but they remain poorly understood. Although the main mode of maintenance of ZIKV in nature is supposed to be the primate-mosquito-primate transmission cycle, evidence of inter-vertebrate (congenital, perinatal, sexual, occupational and others) [1, 5–7] and inter-invertebrate contamination routes (vertical and venereal) [8] may help in explaining the high capacity of ZIKV to be transmitted and spread.

## Conclusion

We conclude that together with other modes of transmission, the venereal route in *Aedes* mosquitoes might contribute to ZIKV virus maintenance in nature.
